# Effects of School-Based Health-Promoting Activities on Children’s Health: A Pragmatic Real-World Study

**DOI:** 10.3390/nu15153349

**Published:** 2023-07-27

**Authors:** Marla T. H. Hahnraths, Bjorn Winkens, Onno C. P. van Schayck

**Affiliations:** 1 Department of Family Medicine, Care and Public Health Research Institute (CAPHRI), Maastricht University, P.O. Box 616, 6200 MD Maastricht, The Netherlands; onno.vanschayck@maastrichtuniversity.nl; 2 Department of Methodology and Statistics, Care and Public Health Research Institute (CAPHRI), Maastricht University, P.O. Box 616, 6200 MD Maastricht, The Netherlands; bjorn.winkens@maastrichtuniversity.nl

**Keywords:** primary school, health promoting school, children, nutrition, physical activity

## Abstract

More insight into the health effects of scaled-up school-based interventions in real-world settings is vital to sustainably integrate health in all schools. This study investigated the effectiveness of the scaled-up Healthy Primary School of the Future (HPSF) initiative in real-world school contexts on children’s health (behaviours). From 2019 to 2022, eleven Dutch primary schools implemented HPSF-related activities. In 315 children from study years four to six (aged 7–11 years) from these schools, anthropometric measurements were performed, and questionnaires assessing the children’s dietary behaviours and physical activity were administered. COVID-19 greatly limited the implementation of HPSF-related activities. Therefore, the results were compared between schools categorised as medium implementers and schools categorised as low implementers. After correction for baseline, waist circumference in the medium implementer group was significantly higher at one-year follow-up (B = 1.089, *p* = 0.003) and two-year follow-up (B = 1.665, *p* < 0.001) compared with waist circumference in the low implementer group. No significant effects were observed for other outcomes. This study showed hardly any effects of the scaled-up HPSF initiative, mainly due to the limited implementation caused by COVID-19. More research investigating the real-world effectiveness of HPSF and comparable programmes is greatly encouraged to advance the field of school-based health promotion.

## 1. Introduction

Globally, the prevalence of childhood overweight and obesity is increasing, and this trend is also visible in the Netherlands [1,2]. As childhood overweight and obesity are known to track into adulthood and increase the risk of both immediate and long-term health problems, reducing their prevalence is vital [3]. Important causes of overweight and obesity include unhealthy lifestyle behaviours (e.g., unhealthy dietary behaviours and insufficient physical activity (PA)). Because lifestyle habits formed during childhood are likely to persist throughout adulthood, the early development of a healthy lifestyle is expected to result in both immediate and long-term health benefits [4,5,6].

Schools are key environments for health promotion. At school, children from various socioeconomic backgrounds come together during critical developmental years, and teachers have the opportunity to educate children about health and serve as role models [7,8,9]. Over the years, many health-promoting interventions have been developed, implemented, and evaluated with the aim of integrating health in schools [10,11,12,13,14].

The Healthy Primary School of the Future (HPSF) is a school-based health-promoting initiative previously implemented in several Dutch schools. The initiative consists of two core components: the daily provision of a healthy school lunch and the daily implementation of structured PA sessions after lunch. Two full HPSF schools implemented both the lunch and the PA components. Two partial HPSF schools implemented the PA component only, and four schools continued with their regular curriculum, serving as control schools in an efficacy trial [15,16]. Longitudinal analyses revealed significant positive intervention effects on outcomes, such as the children’s body mass index (BMI) z-scores and dietary and PA behaviours [17,18,19]. Following the efficacy trial’s positive results, other Dutch primary schools became interested in implementing HPSF-related activities. This created the opportunity to scale up HPSF, i.e., to implement the initiative, which was previously successful on a relatively small scale in a controlled setting, under real-world conditions into broader practice [20]. Scaling up meant working with a wide variety of schools with unique contexts. Moreover, the schools themselves would be mainly responsible for the implementation of HPSF-related activities, as researchers would have limited control over the situation (unlike during the original HPSF trial [15]). Although multiple studies have investigated the efficacy of school-based health-promoting interventions under relatively controlled conditions [10,13,21], there is less research on the effectiveness of such interventions in real-world situations. Gaining insight into the health effects of scaled-up school-based interventions in diverse settings is thus vital to sustainably integrating health in more schools, which can positively impact the health of many children [22,23].

To evaluate the implementation and effectiveness of the scaled-up HPSF initiative, a follow-up research project involving twelve Dutch primary schools was initiated. The schools were not obliged to implement a clear-cut intervention as they were in the HPSF trial; instead, they had the opportunity to implement activities that fit their contexts. This setup facilitated the development of pragmatic, school-specific interventions, which was hypothesised to stimulate sustainable integration of health in the schools. The present study aimed to investigate whether the previously observed positive intervention effects on children’s health-related outcomes would be retained after scaling up HPSF. In order to achieve this, the study aimed to answer the following research questions:

(1) What are the effects of the implemented health-promoting activities on children’s body compositions (BMI z-score and waist circumference)?
(2) What are the effects of the implemented health-promoting activities on children’s PA and dietary behaviours?

## 2. Materials and Methods

### 2.1. Study Design

This effectiveness evaluation was part of a research project investigating the scaling-up of HPSF using a non-randomised, non-controlled design. As we were specifically interested in the natural implementation and the effectiveness of HPSF in a real-world context, no control group was included, and researchers solely played an observing role. This made it possible to observe the implementation of HPSF-related activities without researchers interfering by forcing some schools to implement a specific set of activities or to implement no activities (in the case of control schools). The study involved twelve primary schools that were all members of one educational board.

### 2.2. Setting

The educational board of the twelve participating schools expressed its desire to implement HPSF-related activities. All schools were located in one rural municipality in the northern part of the province of Limburg, the Netherlands. At baseline, the schools’ pupil numbers varied from 31 to 263 (three schools had <100 pupils). One school provided special needs education. Two schools were in the process of merging at baseline, two schools were planning to relocate to other school buildings, and the construction of a new school building for a third school was ongoing. The educational board’s aim was that all schools would eventually implement a daily healthy school lunch and structured PA sessions; the two main intervention components allocated in the original HPSF trial [15]. However, unlike in the original trial, the road towards implementation was not controlled by researchers. Instead, schools were free to decide whether, when, and to what degree they would implement health-promoting activities. All activities had to fit into at least one of the following categories (developed by the educational board): (i) healthy and sustainable nutrition, (ii) sufficient PA, (iii) sufficient rest and relaxation, and (iv) collaboration. The last two categories were not a prominent part of the original HPSF trial but were included in the present project because the stakeholders underpinned their importance for children’s health and well-being. The schools were mainly responsible for their implementation processes but could ask for assistance from a process coordinator appointed by the educational board. This coordinator organised meetings with directors, managers, and staff members to support the implementation processes. The coordinator had an advising role and never forced schools to implement certain activities. Researchers played an observing role to gain insight into the implementation processes.

### 2.3. Comparison of Schools

As the present study investigated the effectiveness of health-promoting activities under real-world conditions and all participating schools were free to implement activities of their choice, there were no intervention or control groups. The ambition at the start of the study was to divide the schools into categories on the basis of their adherence to four HPSF key points. These key points defined HPSF’s optimal implementation and were formulated by stakeholders involved in the original HPSF trial to facilitate HPSF’s further dissemination. In short, these key points entailed (1) active involvement of various stakeholders (e.g., school staff, children, and parents), (2) taking a school-wide approach, (3) all children engaging in at least one hour of PA every day, and (4) all children consuming a daily healthy lunch at school. More details on the categorisation method based on the HPSF key points are described elsewhere [24].

In contrast to the educational board’s initial ambitions, project implementation mainly concerned small, incidental activities (e.g., small adaptations to the schoolyard). The main reasons for this limited implementation were the COVID-19 pandemic and its related restrictions, a lack of commitment and bottom-up involvement, and a high workload. More in-depth information on implementation and the factors influencing it is reported elsewhere [25]. The limited implementation had implications for data categorisation in the present study, as the previously proposed categorisation method could not be used due to low variation in the number of HPSF key points that schools adhered to. Instead, schools were categorised on the basis of the implemented activities’ intensity. This resulted in the formation of two groups: medium implementers (six schools) and low implementers (five schools) (due to the merging of two schools at the start of the project, eleven schools are included in the remainder of this paper instead of the twelve schools at the start of the project). The project’s effectiveness on various health outcomes was compared between these two groups. An overview of the activities implemented within each school and the corresponding categorisation is reported in Table 1; this overview was previously published elsewhere [25].

### 2.4. Participants

At baseline, all children in study years four to six (internationally comparable to grades two to four) and their parents/caregivers (*n* = 649) were invited to participate in the study through information brochures for parents/caregivers. There were no further inclusion or exclusion criteria. In addition to providing information brochures, researchers visited classrooms to inform children about the study and encourage them to participate. After school, parents had the opportunity to ask the researchers questions. All participants were required to complete an informed consent form, which was signed by both parents/caregivers. The need for ethical approval for the overall research project was waived by the Medical Ethics Committee Zuyderland in Heerlen (METC-Z no. METCZ20190144).

### 2.5. Data Collection Procedures

Measurements were conducted in May–July 2020 (T0), 2021 (T1), and 2022 (T2). In each school, data were collected yearly during one measurement week. Inter-rater variability was minimised by training researchers according to a strict protocol.

#### 2.5.1. Anthropometric Measurements

In line with COVID-19-related restrictions applicable at the time, anthropometric measurements were performed in the schoolyard. Children were measured wearing light clothing and no shoes. All anthropometric measurements were performed twice, and a third measurement was conducted if the difference between the first two measurements exceeded a pre-set limit (weight ≥ 0.2 kg, height ≥ 0.5 cm, waist circumference ≥ 1.0 cm) [15]. Weight was measured to the nearest 0.1 kg (Weighing Scale 803, Seca, Hamburg, Germany) and height was measured to the nearest 0.1 cm (Stadiometer 213, Seca, Birmingham, UK) [15]. Waist circumference was measured with a measuring tape to the nearest 0.1 cm, following the World Health Organisation’s assessment protocol (model 201, Seca, Hamburg, Germany) [26].

#### 2.5.2. Questionnaires

Participating children and one of their parents/caregivers were asked to fill out various questionnaires that were largely similar to those used during the original HPSF trial [15].

*Child questionnaire*: All participating children filled out a digital questionnaire assessing their dietary and PA behaviours. The questionnaire was filled out in class during class hours in the presence of at least one researcher. It took about 30 min to complete the questionnaire, as other aspects (e.g., well-being) were also assessed.

*Child lunch questionnaire*: A digital recall questionnaire containing thirteen questions regarding children’s lunch intake was filled out by all participating children. It was filled out immediately after lunchtime in class in the presence of at least one researcher and took about five minutes to complete.

*Parental questionnaire*: A digital parental questionnaire was used to gather information on parental education level and country of birth and children’s health behaviours. Parents/caregivers of all participating children received the questionnaire, which took about 30 min to complete, as other aspects (e.g., quality of life) were also explored. Parents had approximately two months to complete the questionnaire. Two reminders were sent during this period if the questionnaire was not yet completed.

### 2.6. Measures

#### 2.6.1. Covariates

Children’s age, study year, and sex were collected through the educational board’s database. The parental questionnaire was used to gather information on children’s socioeconomic background and ethnicity. Socioeconomic status (SES) was calculated as the mean of standardised scores on maternal and paternal education level [27]. The mean scores were categorised into low, middle, and high SES scores based on tertiles. Children’s ethnicity was determined by the country of birth of both parents and divided into (1) Western background (including the Netherlands and all other European countries (excluding Turkey), North America, Japan, Indonesia, and Oceania) and (2) non-Western background [28]. If at least one of the parents was born in a non-Western country, the child’s ethnicity was assigned as non-Western.

#### 2.6.2. Outcomes

Information on children’s BMI and waist circumference was gathered through anthropometric measurements. Children’s BMI was assessed by height and weight; age- and sex-specific BMI cut-off points were used to define overweight and obesity [29]. BMI z-scores were calculated using Dutch reference values [30].

The child questionnaire was used to gain insight into children’s PA behaviour. The questionnaire contained ten items derived from the International Physical Activity Questionnaire for Children (IPAQ-C), which has acceptable validity [31,32,33]. Activity scores between 1 and 5 were obtained for each item, after which the mean of these scores was calculated to obtain the total activity summary score (ranging from 1 (low PA) to 5 (high PA)) [31].

Water consumption during school hours was derived from the child questionnaire ranging from never (0) to every day (3). Soft drink consumption during the past week was derived from the parental questionnaire ranging from never (0) to every day (7). A composite score for healthy dietary behaviours was computed from four separate questions. This score was calculated by averaging the weekly consumption (ranging from never (0) to every day (7)) of breakfast consumption and intake of fruit, warm and raw vegetables, and water throughout the day, as reported in the parental questionnaire.

The child lunch questionnaire assessed children’s consumption of certain food types during lunch. The items were summarised into six dichotomous (yes/no) food types: fruits, vegetables, grains (bread and cereals), dairy (milk/yoghurt and cheese), water, and butter. To shed more light on the nutritional value of the children’s lunches, the different food types consumed were summed, and a dichotomous variable was created indicating whether children consumed at least two of the food types during lunch.

### 2.7. Data Analysis

Data were analysed using IBM SPSS Statistics for Windows (version 27.0, IBM Corp, Armonk, NY, USA). Pearson’s chi-square tests and independent-samples *t*-tests were conducted to compare the participants’ observed baseline characteristics, i.e., age, study year, sex, SES, ethnicity, BMI z-score, waist circumference, and PA and dietary behaviours, between the medium and low implementer group. Linear mixed model analyses were used to assess the longitudinal intervention effects on children’s BMI z-score, waist circumference, and PA and dietary behaviours; logistic mixed models were used for binary outcomes. Since measurements were repeated within the same group of participants, a two-level model with measurements as the first level and participants as the second level was used. The model’s fixed part consisted of group (medium/low implementers), time (T0/T1/T2), the interaction term of group and time, and the covariates sex (boy/girl), study year at baseline (four/five/six), SES (low/middle/high), and children’s BMI z-score at baseline. In the analyses of children’s BMI z-score, weight status at baseline (underweight/normal weight/overweight) was included instead of BMI z-score at baseline, as this was already corrected for by the model (baseline was included as outcome measure). An unstructured covariance structure for repeated measures was used. Since this model used a likelihood-based approach for missing outcome data, assuming missing at random (MAR), no (multiple) imputation was required. For all analyses, a two-sided *p*-value of ≤0.05 was considered statistically significant. Categorical outcomes (lunch intake outcomes) resulted in odds ratios (ORs) with corresponding 95% confidence intervals.

## 3. Results

### 3.1. Demographic Characteristics

Of the 649 children from study years four to six at baseline, 315 (48.5%) handed in a completed informed consent form to be included in the study. The schools had a median of 31 participating children at baseline (25th percentile = 10; 75th percentile = 39). The parents of 287 children (91.1%) filled out the parental questionnaire at least once. A detailed overview of the included participants at each time point can be found in Supplementary Figure S1. Table 2 provides an overview of the sample’s baseline characteristics.

### 3.2. Effects on Body Composition

**Figure 1:**
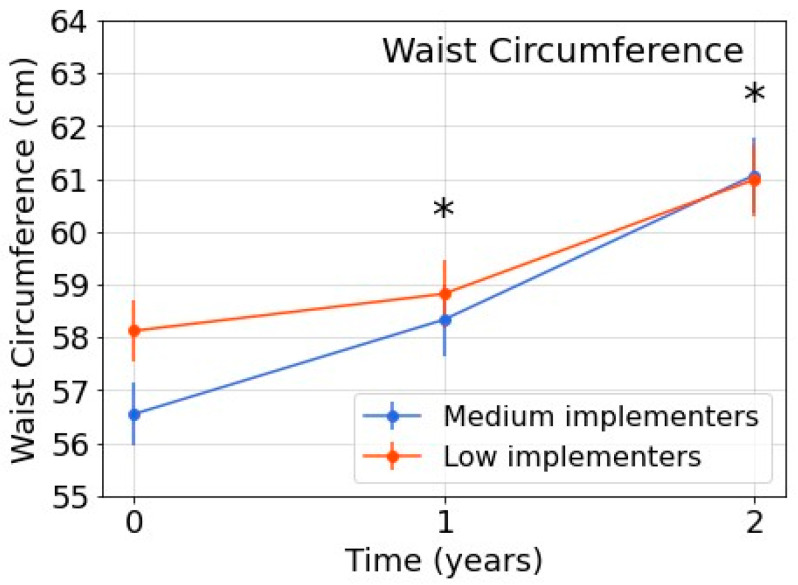
Estimated means of children’s waist circumference at T0–T2. Note: All analyses included sex, study year at T0, SES, and BMI z-score at T0 as fixed factors. * Significant (*p* ≤ 0.05) difference between medium and low implementers after correction for baseline.

For BMI z-score, the interaction between group and time was not significant (*p* = 0.214), showing no significant difference in the change in BMI z-score over time between medium and low implementers (Table 3). For waist circumference, interaction between group and time was significant (*p* < 0.001). At both T1 and T2, the increase from baseline in waist circumference in the medium implementer group was significantly higher than the increase in the low implementer group (Table 3, Figure 1). Descriptive data regarding the observed BMI z-score and waist circumference at T0–T2 can be found in Table S1.

### 3.3. Effects on PA and Dietary Behaviours

The interaction between group and time was not significant for any outcomes related to PA and dietary behaviours, meaning that the change in PA and dietary behaviours over time did not significantly differ between medium and low implementers (Table 3 and Table 4). Descriptive data regarding the observed PA and dietary behaviours at T0–T2 can be found in Table S1.

## 4. Discussion

The present study investigated the effectiveness of the scaled-up HPSF initiative in several real-world primary school contexts. Despite HPSF’s positive health impact observed in the original efficacy trial [17,18,19], only limited effects of the scaled-up initiative could be detected in the present study. However, this does not diminish HPSF’s potential health impact. The lack of observed effects can be attributed to the limited implementation of health-promoting activities. Important reasons for this limited implementation were restrictions related to the COVID-19 pandemic. Due to national safety regulations, schools were forced to close on several occasions and had to cope with numerous sudden changes in their environment. This greatly limited schools’ ability and capacity to work on the project. Further elaboration on the negative impact of the COVID-19 pandemic on project implementation can be found elsewhere [25]. Considering the challenges caused by the COVID-19 pandemic, the limited project implementation and consequent minimal effects are no surprise. Unfortunately, this means that it remains unknown what the effectiveness of the scaled-up HPSF initiative would have been in the absence of the COVID-19 pandemic, in which case schools would have had the opportunity to implement more extensive health-promoting activities.

The significant increase in waist circumference in medium implementers compared with low implementers at both T1 and T2 is striking. Differences in the measurement period could not have influenced this observed trend, as follow-up measurements were performed after exactly one and two years. Furthermore, inter-rater variability was minimised by training researchers to follow a strict protocol and checking the collected data for potential errors. Also, selective drop-out influencing the results was ruled out, as drop-out was minimal (Supplementary Figure S1). At baseline, waist circumference in the medium implementer group was significantly lower than that in the low implementer group. Potentially, the higher increase in waist circumference observed in the medium implementer group over time represented a regression to the mean. There are no commonly accepted reference values for waist circumference in Dutch children. However, a comparison of the present study’s waist circumference values with reference values from Fredriks et al. [34] supports the regression to the mean hypothesis, as the baseline mean waist circumference of medium implementers was lower than the reference value reported by Fredriks et al. for children of comparable age [34].

### 4.1. Strengths and Limitations

The present study is one of the few studies that have investigated the effectiveness of a scaled-up school-based health-promoting initiative in a real-world context, and therefore, it has the potential to provide valuable information for researchers, policymakers, and other stakeholders [22,23,35,36]. However, the project’s pragmatic nature and the limited researcher involvement (researchers solely acted as observers and had no influence on project implementation) made the project and research vulnerable to external influences. The study’s non-randomised nature can be seen as a limitation. However, including schools on the basis of their willingness to participate was a deliberate choice, as it reflects the real-world process of school-based health promotion. Not including a control group in the study (all participating schools implemented health-promoting activities) followed the same reasoning.

At the project’s start, a strong difference in the intensity of project implementation between the schools was expected, which could subsequently serve as a way to categorise and compare schools. However, limited implementation in all schools restricted the ability to create distinct categories and compare schools using the method previously described elsewhere [24]. Instead, schools were categorised into medium and low implementer groups. The small difference in the intensity of the implemented activities between these groups might have contributed to the lack of observed effects. Furthermore, as there was no standardised intervention allocated, all schools implemented their own set of health-promoting activities. Categorising schools into two groups meant that schools implementing (slightly) different activities were combined into the same group, and using a different combination of schools within the groups could have potentially led to different results. However, it was argued that the activities implemented by schools combined in one group were comparable and that the utilised categorisation was, therefore, acceptable. Furthermore, analyses using a different categorisation with three groups (high, medium, and low implementers) showed comparable results, which led to the conclusion that the categorisation of schools had limited influence on the study’s results.

The generalisation of the study’s results should be performed with caution, especially considering the sample’s low ethnic diversity and the fact that all schools were from one educational board and located in one municipality.

Subjective measurements, such as the questionnaires used, might lead to socially desirable answers [37]. To minimise the risk of bias, participants were informed about confidentiality and the fact that there were no right or wrong answers.

### 4.2. Implications for Research and Practice

As the present effectiveness evaluation was largely influenced by the COVID-19 pandemic and its related restrictions, it would be beneficial to perform a comparable study in the absence of a global pandemic to provide more insight into HPSF’s effectiveness when schools have the opportunity to implement more extensive health-promoting activities. Furthermore, considering the fact that previous research on the effectiveness of scaled-up interventions often revealed lower intervention effects than those that were previously observed in efficacy trials, successfully scaling-up interventions remains a challenge [36,38,39]. Therefore, it is vital that future research does not merely focus on the efficacy of school-based health-promoting programmes in relatively controlled settings but moves to the investigation of the effectiveness of these programmes when they are implemented in diverse real-world settings. This could further advance the evidence base and provide valuable information for intervention developers, policymakers, and other stakeholders.

Furthermore, it should be noted that children’s health behaviours are not only influenced by the school environment; other settings, such as the home environment, also play an important role [40,41,42]. Including intervention components targeting the home setting (e.g., family-based activities) is therefore advised to maximise school-based health-promoting programmes’ impact.

## 5. Conclusions

The present study showed hardly any effects of the scaled-up HPSF initiative in real-world school contexts. This minimal effectiveness can be attributed to the limited implementation of health-promoting activities due to the COVID-19 pandemic and its related restrictions. To better inform intervention developers, policymakers, and other stakeholders, more research on the effectiveness of HPSF and other school-based health-promoting programmes in diverse, real-world settings is needed [38].

## Figures and Tables

**Table 1:** Activities implemented in the various schools at the end of data collection and subsequent categorisation into medium and low implementers [25].

Medium Implementers				
School	Healthy and Sustainable Nutrition	Sufficient PA	Sufficient Rest and Relaxation	Social Involvement
1	Daily provision of FV items	(Limited) usage of PA floor for toddlers and preschoolers	Incidental yoga lessons provided by pedagogical employee	N/A
3	- Daily provision of FV items - Pilot to investigate healthy lunch provision (not integrated yet)	- Various staff workshops related to PA integration - Increased amount of education provided outdoors - Adaptation schoolyard (more active elements) - Pilot to investigate structured PA sessions during lunch break time (not integrated yet)	N/A	- Support from volunteers during lunch pilot and FV provision - Support from local companies to provide lunch during pilot
4	Daily provision of FV items	- Integration of an additional 20 min of PA every day (using certified method) - Usage of specific game consoles for outside play	N/A	N/A
6	- Daily provision of FV items - Various activities focused on healthy nutrition (e.g., family food vlogs, classroom-based quizzes, family food experience) - Introduction of new school-wide dietary policy	- Adaptation schoolyard (more active elements) - Integration of PA in curriculum	N/A	Active role for pupils’ voice group and parents in nutrition-related plans
9	- Daily provision of FV items - Daily provision of dairy serving - Expansion of school garden	Integration of an additional 20 min of PA every day (using certified method)	Development of relaxation spaces throughout the school	Active role for volunteers in maintaining school garden
11	Daily provision of FV items	Integration of an additional 20 min of PA every day (using certified method)	N/A	N/A
**Low Implementers**				
2	N/A	- Various staff workshops and information evenings for parents related to PA integration in education - Adaptation schoolyard (more active elements)	Provision of incidental yoga lessons in kindergarten	N/A
5	N/A	N/A	N/A	N/A
7	N/A	- Adaptation schoolyard (more active elements) - Staff workshop regarding reflex integration	N/A	N/A
8	Daily provision of FV items	N/A	N/A	N/A
10	N/A	N/A	Usage of certified method to improve classroom atmosphere	N/A

Note: Due to the merging of two participating schools, eleven schools are included in Table 1 instead of the twelve schools that originally participated in the project. Abbreviations: PA, physical activity; FV, fruit and vegetable; N/A, not applicable.

**Table 2:** Characteristics of participants at baseline (T0) (*n* = 315).

	Total			Medium Implementers			Low Implementers				
	*n*	%/M	SD	*n*	%/M	SD	*n*	%/M	SD	Χ^2^/*t*-Value	*p*
Sex (% boys) ^1^	315	43.8		143	49.7		172	39.0		3.629	0.057
Age (years)	315	9.19	0.98	143	9.21	1.00	172	9.17	0.96	0.320	0.749
Study year (%) ^1^	315			143			172			3.836	0.147
*Four*	100	31.7		48	33.6		52	30.2			
*Five*	99	31.4		37	25.9		62	36.0			
*Six*	116	36.8		58	40.6		58	33.7			
Ethnicity (% Western) ^1^	283	96.8		131	95.4		152	98.0		1.552	0.213
SES (%) ^1,2^	284			131			153			4.921	0.085
*Lowest tertile*	54	19.0		32	24.4		22	14.4			
*Middle tertile*	86	30.3		39	29.8		47	30.7			
*Highest tertile*	144	50.7		60	45.8		84	54.9			
BMI z-score	315	−0.13	0.89	143	−0.15	0.82	172	−0.10	0.94	−0.462	0.645
Overweight/obese (%) ^1^	315	9.8		143	7.0		172	12.2		2.395	0.122
Waist circumference (cm)	315	57.81	5.96	143	56.84	4.66	172	58.62	6.77	−2.741	0.006 *
PA summary score (1–5)	315	2.98	0.66	143	3.04	0.64	172	2.94	0.67	1.285	0.200
Healthy dietary behaviours (mean days/week) ^3^	256	5.56	0.95	114	5.57	0.96	142	5.56	0.95	0.118	0.906
Soft drink consumption (mean days/week)	256	4.17	2.76	114	4.31	2.74	142	4.06	2.78	0.722	0.471
School water consumption (0–3) ^4^	315	1.33	1.17	143	1.43	1.22	172	1.25	1.13	1.378	0.169
Fruit at lunch (% yes) ^1^	315	34.3		143	34.3		172	34.3		0.000	0.995
Vegetables at lunch (% yes) ^1^	315	25.1		143	24.5		172	25.6		0.051	0.822
Grains at lunch (% yes) ^1,5^	315	93.0		143	92.3		172	93.6		0.202	0.653
Dairy at lunch (% yes) ^1,6^	315	35.2		143	37.1		172	33.7		0.382	0.536
Water at lunch (% yes) ^1^	315	29.5		143	32.9		172	26.7		1.407	0.236
Butter at lunch (% yes) ^1^	315	61.0		143	59.4		172	62.2		0.251	0.616
At least two healthy food groups at lunch (% yes) ^1,7^	315	87.9		143	87.4		172	88.4		0.068	0.795

^1^ Analysed by X^2^ test. ^2^ Due to clustering of SES scores at several scores, the tertile group sizes are unequal. ^3^ Healthy dietary behaviours: composite score for frequency of consumption of breakfast, fruit, vegetables, and water. ^4^ School water consumption ranged from never (0) to daily (3). ^5^ Grains consist of the following items: bread and cereals. ^6^ Dairy consisted of the following items: milk/yoghurt and cheese. ^7^ Items in the healthy food groups included fruit, vegetables, grains, dairy, water, and butter. Abbreviations: M, mean; SD, standard deviation; SES, socioeconomic status; BMI, body mass index; PA, physical activity. * Significant (*p* ≤ 0.05) difference between medium and low implementers.

**Table 3:** Estimated intervention effects at T1 and T2 on body composition and PA and dietary behaviours.

		Medium Implementers vs. Low Implementers	
		B (95% CI)	*p*
BMI z-score	**T1–T0**	−0.052 (−0.135; 0.030)	0.210
	**T2–T0**	0.013 (−0.083; 0.108)	0.793
Waist circumference (cm)	**T1–T0**	1.089 (0.377; 1.801)	0.003 *
	**T2–T0**	1.665 (0.774; 2.556)	<0.001 *
PA summary score (1–5)	**T1–T0**	−0.146 (−0.294; 0.002)	0.053
	**T2–T0**	−0.133 (−0.342; 0.075)	0.209
Healthy dietary behaviours (days/week)	**T1–T0**	−0.070 (−0.251; 0.110)	0.444
	**T2–T0**	−0.018 (−0.224; 0.188)	0.863
Soft drink consumption (days/week)	**T1–T0**	0.106 (−0.789; 1.002)	0.815
	**T2–T0**	−0.359 (−1.232; 0.514)	0.418
Water consumption at school (0–3)	**T1–T0**	−0.148 (−0.433; 0.137)	0.307
	**T2–T0**	−0.224 (−0.617; 0.169)	0.263

Note: Analysed by linear mixed model analyses. All analyses included sex, study year at T0, SES, and BMI z-score at T0 or weight status at T0 (only for BMI z-score) as fixed factors. B = estimated group effect in terms of T1–T0 or T2–T0 based on linear mixed model analysis. Abbreviations: BMI, body mass index; PA, physical activity; CI, confidence interval. * Significant (*p* ≤ 0.05) difference between medium and low implementers.

**Table 4:** Estimated intervention effects at T1 and T2 on lunch outcomes.

		Medium Implementers vs. Low Implementers	
		OR (95% CI)	*p*
Fruit (% yes)	**T1–T0**	1.449 (0.771; 2.724)	0.249
	**T2–T0**	1.096 (0.533; 2.252)	0.803
Vegetables (% yes)	**T1–T0**	1.752 (0.948; 3.236)	0.073
	**T2–T0**	1.169 (0.584; 2.337)	0.658
Grains (% yes)	**T1–T0**	1.244 (0.338; 4.574)	0.742
	**T2–T0**	1.830 (0.486; 6.888)	0.370
Dairy (% yes)	**T1–T0**	0.829 (0.490; 1.402)	0.483
	**T2–T0**	1.148 (0.657; 2.006)	0.626
Water (% yes)	**T1–T0**	0.915 (0.501; 1.670)	0.771
	**T2–T0**	0.946 (0.507; 1.765)	0.861
Butter (% yes)	**T1–T0**	1.086 (0.629; 1.877)	0.766
	**T2–T0**	1.002 (0.567; 1.770)	0.995
At least two healthy food groups during lunch (% yes)	**T1–T0**	0.680 (0.257; 1.802)	0.437
	**T2–T0**	1.144 (0.408; 3.209)	0.798

Note: Analysed by Generalised Estimating Equations. All odds ratios were adjusted for sex, study year at T0, SES, and BMI z-score at T0. Abbreviations: OR, odds ratio; CI, confidence interval.

## Data Availability

The data presented in this study are available upon request from the corresponding author. The data are not publicly available.

## Supplementary Materials

The following supporting information can be downloaded at: https://www.mdpi.com/article/10.3390/nu15153349/s1, Figure S1: Flowchart of study participation; Table S1: Observed outcomes at the various time points (T0–T2).
