# Supplementary material for: Effects of School-Based Health-Promoting Activities on Children’s Health: A Pragmatic Real-World Study
Source: Nutrients. 2023 Jul 27;15(15):3349. doi: 10.3390/nu15153349 (PMC10420810; doi:10.3390/nu15153349)
Supplement: Supplementary file 1 [file nutrients-15-03349-s001.zip › Supplementary Table S1. Observed outcomes at the various time points (T0-T2)..pdf]

Supplementary Table S1. Observed outcomes at the various time points (T0-T2).

Table S1. Observed outcomes at the various time points (T0-T2).

	**T0**					
	**Medium Implementers**			**Low Implementers**		
	**n**	**% / M**	**SD**	**n**	**% / M**	**SD**
BMI z-score	143	-0.15	0.82	172	-0.10	0.94
Waist circumference (cm)	143	56.84	4.66	172	58.62	6.77
PA summary score (1-5)	143	3.04	0.64	172	2.94	0.67
Healthy dietary behaviours (days/week) [1]	114	5.57	0.96	142	5.56	0.95
Soft drink consumption (days/week)	114	4.31	2.74	142	4.06	2.78
Water consumption at school (0-3) [2]	143	1.43	1.22	172	1.25	1.13
Fruit (% yes)	143	34.3		172	34.3	
Vegetables (% yes)	143	24.5		172	25.6	
Grains (% yes) [3]	143	92.3		172	93.6	
Dairy (% yes) [4]	143	37.1		172	33.7	
Water (% yes)	143	32.9		172	26.7	
Butter (% yes)	143	59.4		172	62.2	
At least two healthy food groups during lunch (% yes) [5]	143	87.4		172	88.4	
	**T1**					
	**Medium Implementers**			**Low Implementers**		
	**n**	**% / M**	**SD**	**n**	**% / M**	**SD**
BMI z-score	137	-0.14	0.83	168	-0.03	0.97
Waist circumference (cm)	137	58.49	5.64	168	59.40	6.68
PA summary score (1-5)	137	3.19	0.58	168	3.25	0.59
Healthy dietary behaviours (days/week) [1]	90	5.38	0.94	121	5.56	0.90
Soft drink consumption (days/week)	90	4.07	2.67	121	3.63	2.88
Water consumption at school (0-3) [2]	137	1.47	1.13	168	1.40	1.16
Fruit (% yes)	137	31.4		168	26.8	
Vegetables (% yes)	137	31.4		168	23.8	
Grains (% yes) [3]	137	94.2		168	93.5	

Table S1. *Cont.*

	Medium Implementers			Low Implementers		
	**n**	**% / M**	**SD**	**n**	**% / M**	**SD**
Dairy (% yes) [4]	137	39.4		168	39.3	
Water (% yes)	137	38.0		168	31.0	
Butter (% yes)	137	59.1		168	60.7	
At least two healthy food groups during lunch (% yes) [5]	137	86.1		168	88.7	
**T2**						
	**Medium Implementers**			**Low Implementers**		
	**n**	**% / M**	**SD**	**n**	**% / M**	**SD**
BMI z-score	136	-0.10	0.88	166	-0.05	0.98
Waist circumference (cm)	136	61.12	5.94	166	61.57	7.11
PA summary score (1-5)	136	3.12	0.62	166	3.17	0.65
Healthy dietary behaviours (days/week) [1]	82	5.45	0.90	119	5.52	0.84
Soft drink consumption (days/week)	82	3.35	2.61	119	3.43	2.70
Water consumption at school (0-3) [2]	136	1.63	1.20	166	1.73	1.17
Fruit (% yes)	136	22.8		166	23.5	
Vegetables (% yes)	136	19.9		166	19.9	
Grains (% yes) [3]	136	94.9		166	92.8	
Dairy (% yes) [4]	136	48.5		166	38.0	
Water (% yes)	136	44.9		166	38.6	
Butter (% yes)	136	55.1		166	56.6	
At least two healthy food groups during lunch (% yes) [5]	136	89.7		166	86.7	

[1] Healthy dietary behaviours is a composite score for frequency of consumption of breakfast, fruit, vegetables, and water.
[2] School water consumption ranges from never (0) to daily (3).
[3] Grains consists of the items: bread and cereals.
[4] Dairy consists of the items: milk/yoghurt and cheese.
[5] Items in the healthy food groups include: fruit, vegetables, grains, dairy, water, and butter.
Abbreviations: M, mean; SD, standard deviation; SES, socioeconomic status; PA, physical activity.
