# Supplementary material for: Effects of School-Based Health-Promoting Activities on Children’s Health: A Pragmatic Real-World Study
Source: Nutrients. 2023 Jul 27;15(15):3349. doi: 10.3390/nu15153349 (PMC10420810; doi:10.3390/nu15153349)
Supplement: Supplementary file 1 [file nutrients-15-03349-s001.zip › Supplementary Figure S1. Flowchart Study Participation.pdf]

Supplementary Figure S1. Flowchart Study Participation.

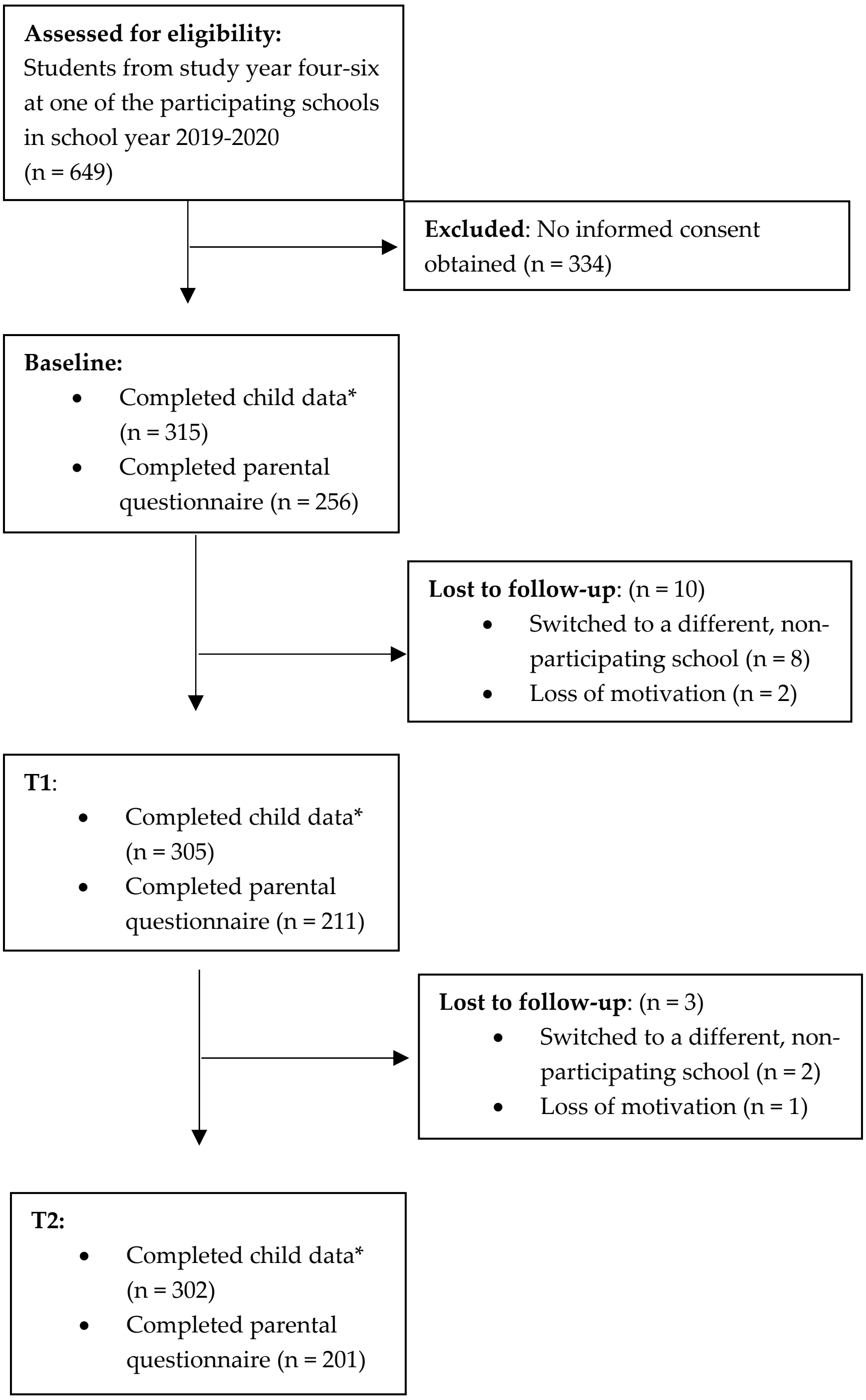

Figure S1. Flowchart Study Participation.
*Child data concerns anthropometric measurements and child-reported questionnaires.
